# Facial Reactivity to Emotional Stimuli is Related to Empathic Concern, Empathic Distress, and Depressive Symptoms in Social Work Students

**DOI:** 10.1177/00332941231181027

**Published:** 2023-07-03

**Authors:** Pierrich Plusquellec, Kaylee Smart, Vincent Denault

**Affiliations:** Centre for Studies in Nonverbal Communication Sciences, Research Centre, Montreal Mental Health University Institute, Montreal, Canada; School of Psychoeducation, University of Montreal, PO Box 6128, Centre-ville-STN, Montreal, QC, H3C 3J7, Canada; School of Psychoeducation, University of Montreal, PO Box 6128, Centre-ville-STN, Montreal, QC, H3C 3J7, Canada; Centre for Studies in Nonverbal Communication Sciences, Research Centre, Montreal Mental Health University Institute, Montreal, Canada; Department of Educational and Counselling Psychology, McGill University, 3700 McTavish Street Montreal, Quebec H3A 1Y2, Canada

**Keywords:** emotional contagion, depressive symptoms, emotional empathy, facial expression, emotional regulation

## Abstract

Helping professionals are exposed daily to the emotional burden of their vulnerable clients and are at risk of unconscious emotional contagion that may lead to stress and emotional distress. Being aware of their own susceptibility to emotional contagion, however, can improve their well-being. This study aimed to propose an objective measure of emotional contagion, complementary to the Emotional Contagion Scale, and to evaluate its construct and predictive validity. To do so, we turned to FACET, an automatic facial coding software using the Facial Action Coding System, to measure participants’ facial expressions as they watched movie clips eliciting specific emotional responses. Results show that both tools to measure emotional contagion (objective and self-reported) are complementary, but they do not measure the same psychosocial constructs. Also, the new objective measure of emotional contagion seems to predict emotional empathy and the risk of developing depressive symptoms among this study’s participants.

## Introduction

Helping professionals are exposed daily to the emotional burden of their vulnerable clients and are at risk of emotional contagion (Petitta et al., 2017; Siebert et al., 2007), as well as stress (Cushway & Tyler, 1996) and burnout (Bakker et al., 2000). Among helping professionals, those beginning their careers are likely more vulnerable given their lack of experience and, sometimes, their idealistic expectations of the profession (Skovholt & Ronnestad, 2003). It is, for example, more difficult for young professionals to understand and regulate the emotional states of their vulnerable clients, as they must first be able to understand and regulate their own (Skovholt & Ronnestad, 2003). In other words, practitioners must be aware, understand, regulate, and express their emotions in order to maintain the therapeutic relationships, while avoiding being emotionally overwhelmed by their clients’ emotions. And students are at risk at the onset of their academic journey. Awareness of their vulnerabilities is thus crucial for their psychological health and future careers.

A self-reporting tool for measuring one’s vulnerability to others’ emotions exists: the Emotional Contagion Scale (ECS; Doherty, 1997). Although this tool was validated in various populations (Lo Coco et al., 2013; Lundqvist, 2006; Riess, 2022), it has limitations, notably due to the nature of self-report questionnaires and their potential bias (e.g., Sato & Kawahara, 2011). Not to mention self-report questionnaires require a certain capacity of introspection and self-awareness. For example, when measuring stress, participants' individual characteristics, such as their personality, ability to regulate emotions, and level of social desirability, can lead to discrepancies between their subjective and objective responses to stress (Campbell & Ehlert, 2012). Therefore, researchers expressed the need to develop a more standardized and objective (rather than suggestive) instrument, specifically for measuring psychosocial constructs such as stress (Campbell & Ehlert, 2012; Pisanski et al., 2018). The same holds for measuring emotional contagion.

With the Emotional Contagion Scale, participants must be able to identify their level of vulnerability to emotions of others. To do so accurately, participants must have consciously experienced such emotions. But this type of emotional awareness is variable in the general population (Ciarrochi et al., 2003), something that has to be considered when devising a method for assessing vulnerability to emotional contagion. Yet, there is currently no standardized and objective instrument to measure one’s vulnerability to others’ emotions. This study offers an objective and replicable tool to measure emotional contagion and to evaluate its construct and predictive validity.

### Emotional Contagion

Emotional contagion is defined as “the tendency to automatically mimic and synchronize facial expressions, vocalizations, postures, and movements with those of another person’s and, consequently, to converge emotionally” (Hatfield et al., 1994), p. 5). This tendency differs from one person to another. That is, some individuals are more sensitive to others’ emotional expressions (Doherty, 1997; Siebert et al., 2007; Verbeke, 1997). As a result, those individuals tend to unconsciously pick up on others’ nonverbal emotional cues more easily. If these people lose control or are unaware of the effects of emotional contagion, they may experience empathic distress as they are more vulnerable to the negative emotions of others (Petitta et al., 2017).

#### Mechanisms of Emotional Contagion

The automatic imitation of nonverbal cues refers to two or more individuals interacting and behaving identically at the same time (Chartrand & Lakin, 2013; Tiedens & Fragale, 2003). The automatic imitation of facial expressions, for example, refers to facial mimicry, which can be described as the involuntary copying of the facial muscles’ contractions of another (Goldman & Sripada, 2005; White & Argo, 2011). Facial mimicry is observed in infants from their earliest days of birth (Field et al., 1982; Haviland & Lelwica, 1987) and continues to be observed throughout childhood (Beall et al., 2008) and adulthood (Hess & Blairy, 2001). However, to some authors, facial mimicry does not prove that both individuals are having the same emotional experience (Prochazkova & Kret, 2017). While the affective component is activated during the involuntary imitation, the psychological component, associated with a shared emotional experience, is not necessarily activated (Prochazkova & Kret, 2017). Thus, the automatic imitation of nonverbal cues alone is insufficient to generate emotional contagion.

Some studies measuring emotional contagion suggest that copying nonverbal emotional cues would help in improving recognition of emotional states through the facial feedback process (Davis et al., 2015; Dzokoto et al., 2014). Indeed, facial feedback would elicit an emotional experience consistent with the expressed emotion (Buck, 1980; Goldman & Sripada, 2005). Not to mention, when an individual experiences an emotion, increasing the facial expression related to the emotion could increase the intensity of that emotion (Ekman et al., 1980; Ekman & Friesen, 1976), whereas repressing the facial expression could decrease the emotional intensity (Soderkvist et al., 2018). In other words, facial feedback could both initiate and regulate an emotional experience (Adelmann & Zajonc, 1989; Marsh et al., 2019). However, these last results are subject to criticism. In Coles et al. (2019), for example, a meta-analysis of 138 studies only partially supported the facial feedback process, which was further questioned by a recent multi-lab study (Coles et al., 2022). According to the authors, facial feedback could influence the recognition of others’ emotions, but only in certain contexts and for certain emotions. And other authors suggest that facial feedback could help in recognizing others’ emotions only when the observer’s emotional experience is considered to be related to the emotional expression perceived in others, and when emotions such as joy and anger are involved (Clore et al., 2018; Coles et al., 2019; Laird, 1984).

In addition to improving recognition of emotional states, copying nonverbal emotional cues may influence the observer’s own emotional experience (Prochazkova & Kret, 2017). Affective empathy, that is, feeling the same emotions as others after perceiving their emotional facial expressions, is associated with the amount of facial muscle activity (Prochazkova & Kret, 2017; Rymarczyk et al., 2016). In other words, the higher an individual’s level of affective empathy is, the more sensitive the individual is to emotional facial expressions of others and, in turn, is likely to display those emotional facial expressions. As a result, by recognizing and replicating emotional facial expressions of others, facial mimicry allows for a greater understanding of others’ affects (Hess et al., 2014).

#### Consequences of Emotional Contagion

Regardless of the mechanism for emotional contagion, vulnerability to others’ emotions can result in adverse consequences (Buchanan et al., 2012; Siebert et al., 2007). For example, Siebert (2004) has shown that social workers facing symptoms of their depressed or distressed clientele on a daily basis show rates of depression that are three times higher than the general population, and emotional contagion may be a contributing factor. That is, being unaware of this tendency while having difficulty distancing themselves from their clienteles’ emotional states puts social workers at risk of experiencing the depressive or distressing symptoms they perceive (Siebert et al., 2007). In other words, because they work with vulnerable clienteles experiencing negative emotions, such as fear, anxiety, and distress, healthcare professionals, and, specifically, social workers are at risk of experiencing the adverse consequence of emotional contagion (Bakker et al., 2000; Petitta et al., 2017; Sánchez-Moreno et al., 2015). This is why researchers emphasized the importance of examining emotional contagion among professionals who help distressed clienteles (Sánchez-Moreno et al., 2015; Siebert et al., 2007; Verbeke, 1997). This issue is all the more important when considering students in training, as they have their first clinical experiences, but do not necessarily have the skills to be aware of their vulnerabilities or to cope with them (Schwartz-Mette, 2009).

##### Empathy

For healthcare providers, empathy is a central relational pattern (Neumann et al., 2011; Riess, 2022), and it is highly encouraged among social work students. Empathy can be considered as “an induction process that reflects an innate ability to perceive and be sensitive to the emotional states of others, which can be, but not necessarily is, coupled with a motivation to care for their well-being” (Decety et al., 2016). Several theoretical models explain the emergence of empathy from the mechanism of emotional contagion, including the perception-action model (Preston & de Waal, 2002) and the neurocognitive model of emotional contagion (Prochazkova & Kret, 2017).

Although empathy is associated with emotional contagion, the two constructs are different because cognitive components are added to the process, including cognitive strategies akin to regulation such as the ability to distinguish self from others or to put the situation experienced by the other into perspective (Klimecki & Singer, 2013). In other words, it is essential for social workers to be empathic to be aware that they are experiencing emotions and that these emotions are not their own. This distinction requires social workers to be able to efficiently identify and regulate their emotions.

##### Emotional Regulation

Emotional regulation can be defined as “a process by which the individual modulates his or her emotions to respond appropriately to the demands of the environment” (Côté et al., 2013). According to Gratz and Roemer (2004) model, emotional regulation is a multidimensional construct, including four distinct skills: “(1) awareness and understanding of emotions (2) acceptance of emotions (3) the ability to engage in goal-directed behavior, and refrain from impulsive behavior, when experiencing negative emotions; and (4) access to emotion regulation strategies perceived as effective.” When helping professionals remain unaware of their vulnerability to others’ emotions and have emotional regulation deficits, including deficits in emotional awareness, they are more at risk of experiencing the adverse consequences of emotional contagion.

#### Emotional Contagion and Emotional Awareness

Emotional awareness is particularly relevant when considering emotional contagion and the positive or negative consequences of this tendency. Awareness of one’s own emotions involves “paying attention to and reflecting on automatically generated body experiences” (Lane & Smith, 2021), even those generated by imitation, and helps people infer what the interaction meant to them, and what specific needs the interaction generated. Awareness of physical sensations, also called interoception, could be considered an initial step in emotional awareness. In a recent systematic review, Pinna and Edwards (2020) suggest that high interoception was predictive of effective downregulation of negative emotions and handling of social uncertainty, something that could be considered the daily life of social work students. In fact, interoception is increasingly studied in relation to empathy and research findings suggest a positive association between interoception and self-other distinction (Tajadura-Jimenez & Tsakiris, 2014; Tsakiris et al., 2011), which can be considered as an essential skill for social workers. A higher ability in self-other distinction is related to less affect sharing in social situation, i.e. emotional contagion (de Vignemont & Singer, 2006). Interestingly, interoception seems to have a complex relationship with the Emotional Contagion Scale, per se. In a study with 82 participants, interoception was positively associated with emotional contagion for negative emotions but not positive emotions of others. This result was found only in the female participants, which could be caused by higher level of interoception in male than in female participants (Lischke et al., 2020). In other words, high emotional awareness through bodily sensations would protect from emotional contagion, but only in women.

#### Measuring Emotional Contagion

As previously mentioned, emotional contagion is usually measured by the ECS. The self-report questionnaire was developed by Doherty (1997) to measure an individual’s susceptibility to align with the emotions expressed by others. The items in the questionnaire were designed to assess participants’ responses to five core emotions (joy, love, fear, anger, and sadness) and attention to the emotions of others. The items refer to situations in which a person would be exposed to the emotions of others (e.g., “Being with a happy person cheers me up when I am not feeling well,” “I get irritated when I am surrounded by angry people,” or “Hearing the shrill cry of a terrified child in the dentist’s waiting room makes me nervous”). However, because it is a self-report questionnaire, it relies solely on respondents’ perception and introspective abilities to determine their degree of vulnerability to emotional contagion. This tool therefore assumes that participants are aware of their emotional reactivity to others’ emotions, which is not always the case. Low emotional awareness put people at greater risk of having difficulties in regulating their emotions and, therefore, of developing psychopathology (Subic-Wrana et al., 2014), whereas a good understanding and awareness of one’s emotional states are associated with well-being (Duran et al., 2004). This point highlights an important bias, in addition to biases inherent in a self-report nature of the questionnaire. Although the ECS is validated, the participants’ scores might be dependent on the participants’ ability to be emotionally aware. Thus, an objective measure of emotional contagion, complementary to the ECS, would be an asset for determining people’s emotional reactivity to others’ emotions, particularly in people who are less aware of their own emotions.

To objectively measure emotional contagion, and to take into account interoception, heart rate synchronization seems to be an appropriate solution. Research shows, in fact, that synchronization of physiological measurements between two people interacting with each other is positively related to emotional contagion (Park et al., 2019). However, developing a measure based on heart rate synchronization would require having a stimulus person who would always respond in the same way, which is not obvious in laboratory settings. Another way to objectively measure emotional contagion could be through facial expressions, as they provide important indications of the subject’s emotional state (Du et al., 2014). However, until recently, the measurement of facial expressions required the use of electromyography protocols, which is an unfriendly technique and, again, limited to laboratory settings. However, the automatic analysis of facial expressions offered new possibilities (Sikka et al., 2015; McDuff et al., 2016; McDuff & el Kaliouby, 2017).

Thus, to circumvent biases related to self-report questionnaires, and to be able to objectively capture participants’ reactivity to emotions of others, regardless of their emotional awareness abilities, we turned to FACET (Bartlett et al., 2014; Littlewort et al., 2011), an automatic facial coding software using the Facial Action Coding System (Ekman et al., 1980). Recently, researchers have used this type of software to examine the relationship between facial responses to emotional stimuli and the participants’ behavioral and temperamental characteristics. For example, Fanti et al. (2017) used FaceReader (Noldus Information Technology, Wageningen, The Netherlands), a software similar to FACET, to examine the facial reactions of young adults with varying levels of emotional insensitivity and impulsive aggressive behaviors when viewing violent and comedy movies. The researchers found that watching violent movies was associated with reduced facial reactions of sadness and disgust in participants with high levels of emotional insensitivity and increased facial reactions of anger in impulsive aggressive participants. In another study, Khvatskaya and Lenzenweger (2016) used the CERT software (Bartlett et al., 2014), a forerunner of FACET, to analyze facial expressions following the viewing of images from the International Affective Picture System in young adults (males and females) with high psychopathic traits compared to those with low psychopathic traits. Results showed that both males and females with high psychopathic traits, compared to those with low psychopathic traits, expressed significantly less emotional congruence when viewing images of negative emotions. This suggested a deficit in motor empathy, the unconscious response to emotional stimuli.

Using FACET to assess the facial activity of students watching a set of video stimuli validated to elicit various emotional responses (Schaefer et al., 2010), this study aims to propose an objective measure of emotional contagion, independent from the respondent’s emotional awareness abilities, and to evaluate its association with the self-reported scale of emotional contagion and its predictive validity. Our hypotheses are that (a) both subjective and objective measures are not associated because of variation in emotional awareness in our sample of participants, (b) our objective measure of emotional contagion will have a better predictive validity than the self-reported because it will encompass more of the emotional contagion construct. For this second hypothesis, we tested whether both emotional contagion measures could predict not only empathy but also the risk of chronic stress and depressive symptoms in our sample after controlling for sociodemographic variables and difficulties in emotion regulation.

## Method

### Participants

A cross-sectional research design was used. Data collection took place at a single measurement time, in fall 2019. The study sample consisted of 56 undergraduate and graduate social work students at the University of Montreal. Women made up the majority of the study sample (85.71%). This was expected because of the representativeness of women in such university programs (Richmond et al., 2015). No financial incentives were used during the recruitment period. The only inclusion criterion was to be enrolled in fall 2019 in one of the programs offered by the School of Psychoeducation of Université de Montreal. These programs are very popular and are therefore contingent. They include a selection process. Most students in these programs will become social workers. No exclusion criteria were considered. The project was approved by the Educational and Psychological Research Ethics Board (CEREP-19-078-D).

### Procedures and Measures

#### Measure of Emotional Contagion

Two tests were used to measure emotional contagion. The first was the ECS (Doherty, 1997). This self-report questionnaire measures inter-individual differences in susceptibility to “catching” the emotions of others. It consists of five subscales of three items each representing contagion of five basic emotions: joy, sadness, love, fear, and anger. Participants respond on a five-point Likert scale ranging from “never” to “always.” Since the multidimensional structure of the ECS is still under debate, we used only the total score (Lo Coco et al., 2013; Lundqvist, 2006; Rueff-Lopes & Caetano, 2012; Wróbel & Lundqvist, 2014).

The second test was a new standardized measurement tool for measuring one’s vulnerability to emotional contagion. While participants viewed movie clips eliciting specific emotional responses - joy, anger, fear, sadness, disgust, surprise, and neutral (Schaefer et al., 2010; see Supplementary Table 1) - their facial expressions were filmed and coded automatically using FACET (Bartlett et al., 2014). FACET provides a measure of evidence value for the seven basic emotions (joy, anger, fear, sadness, disgust, surprise, and neutral). The evidence value represents the probability that the facial expressions associated with these emotions are present and recognized as such by the software. For example, a joy evidence value of +2 means that a human coder would be 100 times more likely to code a facial expression as joy than not as joy. On the other hand, a joy evidence value of −2 means that a coder would be 100 times less likely to code the facial expression as joy. An evidence value of 0 means that there are as many chances that the facial expression will be categorized as joy than not. For our study, the variable used is the maximum evidence value, which is the maximum facial reactivity of the participant when viewing movie clips related to each emotion. For example, we used the maximum evidence value for joy displayed by the participants across the four movie clips chosen to elicit joy. This variable was considered as a proxy to assess the intensity of participants’ congruent facial reactivity to each emotion and, thus, the susceptibility to emotional contagion from the movie clips presented. We chose to use as video stimuli a database of movie clips validated to elicit each emotion (Schaefer et al., 2010). To respect this validity criterion, we did not edit any video segment. As a result, the clips can have different durations (see Supplementary Table 1). For each emotion, the cumulative exposure durations are as follows: Joy (479s), Sadness (383s), anger (255s), fear (648s), disgust (400s), surprise (127s), neutral (41s).

Participants were invited to the lab to view the movie clips. Upon arrival, they filled out the self-reported questionnaires and then completed the task. At the end of the task, a debriefing was conducted to ensure that participants had returned to a baseline emotional level. The video of each participant’s face was recorded from the webcam built into the computer (MacBook Pro 2018) that played the movie clips. The video file was then extracted, transformed into the appropriate video format using TunesKit Free video cutter, and the recordings were analyzed with FACET software from iMotions, Inc.

#### Measure of Empathy

Empathy was assessed by the Interpersonal Reactivity Index (IRI), a 28-item questionnaire with four subscales with good internal consistency ranging from .70 to .81 (Gilet et al., 2013). It is designed to explore emotional and cognitive empathy. Emotional empathy is represented by two subscales, that is, tendency to be affected by the suffering of others (empathic concern) and personal distress related to the reaction to this suffering. Cognitive empathy, on the other hand, is represented by two subscales referring to the ability to understand the other’s point of view (perspective taking) and the ability to project oneself emotionally and actively in the fictitious characters of books, films, plays, etc. (fantasy scale). Considering our objective, we chose to use only the two subscales relative to emotional empathy.

#### Measure of Depressive Symptoms

The manifestation of depressive symptoms was measured using the Beck Depression Inventory (BDI) (Beck et al., 1988). This self-report questionnaire is used to assess the presence and intensity of depression in clinical and nonclinical populations. It features 21 items reflecting different symptoms and attitudes which, separately, can be rated from 0 to 3 in terms of intensity. The French version has been validated in a sample of Canadian students (Bourque & Beaudette, 1982).

#### Measure of Chronic Stress

The Trier Inventory of Chronic Stress (TICS) (Schulz & Schlotz, 1999) was used to measure perceived chronic stress. This self-report questionnaire consists of 57 items that can be grouped into nine different factors: work overload, social overload, pressure to perform, job dissatisfaction, excessive work demands, lack of recognition, social tension, social isolation, and chronic worry. These nine factors can be grouped into two higher-order factors: high demands and lack of satisfaction (Petrowski et al., 2012). Items are rated on a five-point Likert scale ranging from 0 (never) to 4 (very often).

#### Measure of Emotional Regulation

The French version of Difficulties in Emotion Regulation Scale (DERS-F), (Dan-Glauser & Scherer, 2013; Gratz & Roemer, 2004) measures difficulties in emotional regulation. This self-report questionnaire is composed of six dimensions: unacceptance of negative emotions, difficulty engaging in goal-directed behaviors in the presence of negative emotions, difficulty controlling impulsive behaviours in the presence of negative emotions, limited access to emotion regulation strategies perceived as effective, lack of emotional awareness, and lack of understanding of one’s emotions defined as a lack of emotional clarity. It consists of 36 items rated on a six-point Likert scale. One can use the overall score, or the subscale related to each dimension.

### Analytical Strategy

To develop a picture of the participants, a descriptive analysis of the data was conducted to extract the mean and the distribution of each variable. Then, associations between both emotional contagion measurement tools were conducted to evaluate whether the proposed standardized measurement tool really measured what it claims to measure for the social work students. Multiple linear regressions were also conducted to determine how much of the variance in empathy, chronic stress and depressive symptoms could be explained by our independent variables, particularly those derived from this new tool, and the control variables. The simple correlation and multiple linear regression analyses were performed using the Statistical Package for Social Sciences (SPSS) program (version 26, IBM, Inc.), and the minimum statistical probability threshold was set at *p* < 0.05.

## Results

### Description of the Sample

As expected, our sample was predominantly female (85.71%). The average age was 24.89 years (s.d. 4.94, min 20, max 50). The participants were either in undergraduate (42.86%) or graduate (57.14%) studies. Slightly more than half (51.79%) were employed in paid helping professions. Their annual revenues were under $5,000 (14.3%), $5,000-$10,000 (26.8%), $10,000-$15,000 (23.2%), $15,000-$20,000 (12.5%), $20,000-$25,000 (7.1%), $25,000-$30,000 (7.1%), and over $30,000 (8.9%). On a personal level, most students (64.29%) had received counseling services and just over one in five students had a mental health diagnosis (21.43%), including depression (minor or major), social anxiety, adjustment disorder with depressed mood, situational anxiety, generalized anxiety disorder, panic disorder, unspecified anxiety disorder, dysthymic disorder, attention deficit hyperactivity disorder, giftedness, or adjustment difficulties.

Table 1 presents the descriptive statistics for the independent variables (i.e., emotional contagion, objective measurement and self-report), the moderator variable (i.e., emotional regulation), and the dependent variables (i.e., empathy, chronic stress, and manifestation of depressive symptoms).

**Table 1.** Descriptive Statistics of the Independent, Dependent, and Moderate Variables.

	Mean	SD	Min	Max	α^1^
Facial responsiveness to emotion-elicited movie clips					
Max facial responsiveness	1.87	1.29	0.17	5.54	
Max facial responsiveness of joy for « joy » films	2.57	2.32	−3.45	9.51	
Max facial responsiveness of anger for « anger » films	0.95	0.84	−2.09	3.12	
Max facial responsiveness of surprise for « surprise » films	0.15	0.92	−3.39	3.06	
Max facial responsiveness of fear for « fear » films	0.11	0.77	−2.88	1.89	
Max facial responsiveness of disgust for « disgust » films	0.54	0.79	−1.99	2.35	
Max facial responsiveness of sadness for « sadness » films	0.35	0.45	−0.78	1.57	
Max facial responsiveness of neutral for « neutral » films	1.59	0.57	0.16	2.86	
ECS – Emotional contagion scale					
Total score	50.57	8.09	27.00	69.00	0.80
DERS-F – Difficulties in emotion regulation scale					
Total score	78.12	21.13	46.00	139.00	0.94
Nonacceptance of emotional response	12.13	5.60	6.00	28.00	0.94
Difficulties in adopting goal-directed behaviors	15.00	5.18	6.00	24.00	0.93
Difficulties in controlling impulsive behaviors	10.32	4.17	6.00	23.00	0.85
Lack of emotional awareness	14.15	5.02	6.00	25.00	0.85
Limited access to emotion regulation strategies	16.66	5.57	9.00	34.00	0.85
Lack of emotional clarity	9.86	3.22	5.00	17.00	0.80
IRI – Interpersonal Reactivity Index					
Personal distress	24.29	6.84	12.00	39.00	0.80
Empathic concern	38.90	5.17	26.00	49.00	0.66
TICS 2 factors-model – Trier Inventory of Chronic Stress					
High demands	28.89	10.65	12.00	59.00	0.91
Lack of satisfaction	24.96	9.56	8.00	50.00	0.84
BDI – Beck Depression Inventory					
Total score	10.07	7.16	1.00	32.00	0.87

*Note*. SD: standard deviation; Min: Minimum; Max: Maximum.^1^α: Cronbach alpha is provided to give information about the internal consistencies of the scales in the sample.

### Intercorrelations between Variables

Table 2 presents the Pearson correlation coefficients between the different variables. To highlight the most interesting associations, we decided to keep only the total scores of the measurement instruments, except for emotional empathy with two complementary subscales and the variables related to the proposed standardized measurement tool keeping facial responsiveness to each emotion.

**Table 2.** Pearson Correlations Between Outcomes.

	1	2	3	4	5	6	7	8	9	10	11	12	13	14	15	16	17	18	19
1. Age																			
2. Gender	−.10																		
3. Annual revenue	.41^***^	−.04																	
4. Undergraduate/graduate	.28*	.04	.16																
5. Used counseling services	.20	−.12	.01	−.04															
6. Max facial responsiveness	−.15	−.09	.05	−.03	.18														
7. Max facial responsiveness of joy	−.10	.02	.01	.01	.17	.87^***^													
8. Max facial responsiveness of anger	−.03	−.26	−.06	−.05	.34*	.29^*^	.26												
9. Max facial responsiveness of surprise	−.09	−.02	−.24	−.03	.21	.13	.25	.13											
10. Max facial responsiveness of fear	−.10	−.03	−.08	−.02	.23	.45^***^	.47^***^	.23	.66^***^										
11. Max facial responsiveness of disgust	−.03	−.03	.06	.09	.27*	.51^***^	.41^**^	.40^**^	.28^*^	.54^***^									
12. Max facial responsiveness of sadness	.01	−.13	−.09	−.10	.25	.14	.14	.37^**^	.25	.36^**^	.26								
13. Max facial responsiveness of neutral	−.15	.14	.00	−.04	−.04	−.08	.02	−.03	.18	.09	−.12	.21							
14. ECS total score	.18	−.39^**^	.11	−.05	.09	.19	.14	−.05	−.06	−.11	.01	−.09	−.15						
15. DERS-F total score	−.26	−.15	−.15	−.22	.27*	.04	.10	−.08	.32^*^	.12	−.03	.05	.15	.19					
16. IRI – Personal distress	.01	−.20	−.26^*^	.08	.02	−.06	−.02	.03	.36^**^	.08	.01	.20	.28^*^	.31^*^	.45^***^				
17. IRI – Empathic concern	.04	−.38^**^	.06	−.20	.05	−.15	−.22	.03	−.17	−.17	−.15	−.08	−.26	.40^**^	.14	.10			
18. TICS – High demands	−.26	−.09	−.13	−.28^*^	.17	.17	.13	.06	.21	.13	−.02	.02	−.08	.22	.58^***^	.17	.19		
19. TICS – Lack of satisfaction	−.23	−.08	.03	−.30*	.24	.23	.22	.05	.20	.20	.03	.04	−.07	.20	.50^***^	−.01	.21	.92^***^	
20. BDI – Total score	−.22	−.10	−.22	−.17	.34^**^	.26	.25	.24	.30^*^	.17	.22	.10	−.12	.06	.55^***^	.11^*^	.16	.54^***^	.51^***^

*Note.* ^*^p < .05; ^**^p < .01; ^***^p < .001.

Measures of facial responsiveness did not appear to be associated with those of the ECS, indicating that they probably do not assess the same construct. On the other hand, 14 out of 36 possible correlations were significant between variables looking at facial responsiveness. For example, maximal facial responsiveness of fear during films eliciting fear was significantly associated with maximal facial responsiveness of disgust during films eliciting disgust (r_56_ = .54, *p* < .001).

According to the correlation matrix, and as expected, gender was significantly associated with the ECS total score (r_56_ = −.39, *p* < .003), with women having a higher score than men. Gender was also related to empathic concerns (r_56_ = −.38, *p* < .004), with women having higher scores to this scale than men. The level of education (undergraduate vs. graduate) was significantly associated with the levels of chronic stress. Being undergraduate was indeed associated with a higher level of chronic stress due to high demands (r_56_ = −.28, *p* < .04), and with a high level of chronic stress due to a lack of satisfaction (r_56_ = −.30, *p* < .02). Having already used counselling services was related not only to a significant increase in showing a high maximal facial responsiveness to anger (r_56_ = .34, *p* < .01), and disgust (r_56_ = .27, *p* < .04), but also to a risk of having more difficulties in emotion regulation (r_56_ = .27, *p* < .04), and more depressive symptoms (r_56_ = .34, *p* < .01).

As for the ECS, the higher the participants scored on the scale, the higher their levels of empathic concern (r_56_ = .40, *p* < .002), and personal distress were (r_56_ = .31, *p* < .02). The ECS score, but not the facial responsiveness variables, appeared thus to be related to both affective subscales of empathy.

Furthermore, a few associations emerged between the different measures of the proposed standardized measurement tool and other variables. First, students who exhibited a higher peak of facial reactivity when viewing movie clips tended to be the ones who reported having higher levels of depressive symptoms (respectively, r_56_ = 0.26, *p* = .053). Also, the participants who demonstrated surprise while viewing the surprise-inducing movie clips were those who reported having higher levels of depressive symptoms (r_56_ = .30, *p* < .03) as well as more personal distress (r_56_ = .36, *p* < .006) and more difficulties in emotion regulation (r_56_ = .32, *p* < .02).

There were also significant positive associations between the DERS-F total score and one subscale of empathy, both dimensions of the TICS, and the total scores on the BDI. In other words, the more difficulties in emotion regulation a person had, the higher their personal distress (r_56_ = .45, *p* < .001), the greater their risk for chronic stress related to high demands (r_56_ = .58, *p* < .01) and dissatisfaction (r_56_ = .50, *p* < .001), and the greater their depressive symptoms (r_56_ = .55, *p* < .001).

### Multiple Linear Regression

To better understand the relationship between the variables associated with emotional contagion, emotional empathy, chronic stress, and depressive symptoms, five linear regressions were tested. The variables included in each of the models were the same, that is, sociodemographic variables (age, sex, annual revenue, grade, experience of counseling services), the measures of facial responsiveness, the self-reported Emotional Contagion Total Score (ECS), and the self-reported Emotional Regulation Total Score (DERS-F). According to (Austin & Steyerberg, 2015), linear regression models require only two subjects per variables for an adequate estimation of regression coefficients, standard errors, and confidence intervals. Each of our regression models included 15 variables, and our number of subjects was 56. No multicollinearity problems were observed between predictors (Tabachnick & Fidell, 2007). Regressions were run in two stages. First, each regression was run hierarchically to assess the amount of variance that could be explained by the independent variables. Then, a stepwise backward deletion regression method was used to maintain a parsimonious model that included only significant variables to explain the variance of the dependent variables (chronic stress or depressive symptoms).

#### Personal Distress Model

The 11th block predictor in Table 3 explained 38% of the variance in the model [adjusted R2 = .38; F (5.50) = 7.75, *p* < .001]. The more students reported having difficulties with emotion regulation, being more vulnerable to emotional contagion, displaying more neutral facial expression during neutral films, being a graduate (vs. undergraduate), and having a lower revenue, the more they experienced personal distress.

**Table 3.** Hierarchical Regression Results for Personal Distress.

Variable	B	95% CI for B		SE B	β	Adjusted *R*^2^
		LL	UL			
Step 1						.41
Constant	−9.61	−25.84	6.64	8.03		
Age	.30	−.09	.70	.19	.22	
Gender	−.40	−5.34	4.54	2.44	−.02	
Annual revenue	−1.10^*^	−2.04	−.17	.46	−.29^*^	
Level of education	2.71	−.42	5.83	1.55	.20	
Counseling services	−3.51	−7.17	.15	1.81	−.25	
Max facial responsiveness	.60	−2.13	3.32	1.35	.11	
Max facial responsiveness of joy	−.65	−2.05	.76	.70	−.22	
Max facial responsiveness of anger	.77	−1.43	2.97	1.09	.09	
Max facial responsiveness of surprise	1.99	−.38	4.36	1.17	.27	
Max facial responsiveness of fear	−1.32	−4.49	1.85	1.57	−.15	
Max facial responsiveness of disgust	.76	−1.82	3.34	1.29	.09	
Max facial responsiveness of sadness	2.19	−1.60	5.98	1.87	.14	
Max facial responsiveness of neutral	3.15^*^	.31	5.90	1.40	.26^*^	
ECS total score	.26^*^	.05	.48	.11	.31^*^	
DERS-F total score	.14^**^	.05	.23	.04	.43^**^	
Step 11						.38
Constant	−2.64	−14.31	9.02	5.81		
Annual revenue	−1.07^*^	−1.90	−.24	.41	−.28^*^	
Level of education	3.17^*^	.14	6.19	1.50	.23^*^	
Max facial responsiveness of neutral	3.49^*^	.82	6.15	1.32	.28^*^	
ECS total score	.28^**^	.09	.47	.09	.33^**^	
DERS-F total score	.11^**^	.04	.19	.04	.36^**^	

*Note.* ^*^p < .05; ^**^p < .01; ^***^p < .001.

#### Empathic Concern Model

The 11th block predictor in Table 4 explained 28% of the variance in the model [adjusted R2 = .28; F (5.50) = 5.34, *p* < .001]. Having less facial responsiveness and reporting more vulnerability to emotional contagion increased the level of empathic concern.

**Table 4.** Hierarchical Regression Results for Empathic Concern.

Variable	B	95% CI for B		SE B	β	Adjusted *R*^2^
		LL	UL			
Step 1						.17
Constant	34.06^***^	19.49	48.64	7.21		
Age	−.10	−.45	.26	.17	−.09	
Gender	−2.85	−7.29	1.58	2.19	−.19	
Annual revenue	.24	−.59	1.08	.41	.09	
Level of education	1.50	−4.30	1.31	1.39	−.14	
Counseling services	.32	−2.97	3.61	1.63	.03	
Max facial responsiveness	−.73	−3.18	1.72	1.21	−.18	
Max facial responsiveness of joy	−.32	−1.58	.94	.62	−.14	
Max facial responsiveness of anger	.69	−1.29	2.66	.98	.11	
Max facial responsiveness of surprise	−.99	−3.11	1.14	1.05	−.18	
Max facial responsiveness of fear	1.19	−1.66	4.03	1.41	.18	
Max facial responsiveness of disgust	−.79	−3.11	1.52	1.14	−.12	
Max facial responsiveness of sadness	−.41	−3.81	2.99	1.68	−.04	
Max facial responsiveness of neutral	−1.96	−4.50	.59	1.26	−.21	
ECS total score	.21^*^	.02	.41	.10	.34^*^	
DERS-F total score	.02	−.06	.10	.04	.10	
Step 11						.28
Constant	35.54^***^	25.99	45.10	4.76		
Gender	−3.61	−7.27	.06	1.82	−.25	
Level of education	−2.01	−4.39	.37	1.18	−.19	
Max facial responsiveness	−1.03^*^	−1.97	−.09	.47	−.26^*^	
Max facial responsiveness of neutral	−1.89	−4.03	.24	1.06	−.21	
ECS total score	.20^*^	.03	.36	.08	.31^*^	

*Note.* ^*^p < .05; ^**^p < .01; ^***^p < .001.

#### Chronic Stress Model Related to High Demands

The 15th block predictor in Table 5 explained 32% of the variance in the model [adjusted R2 = .32; F (1.54) = 27.36, *p* < .001]. The more students reported having difficulties with emotion regulation, the more they experienced chronic stress related to high demands in their professional and personal lives.

**Table 5.** Hierarchical Regression Results for Chronic High Demand Stress.

Variable	B	95% CI for B		SE B	β	Adjusted *R*^2^
		LL	UL			
Step 1						.28
Constant	5.06	−22.88	33.01	13.83		
Age	−.22	−.89	.45	.33	−.10	
Gender	4.58	−3.92	13.09	4.21	.15	
Annual revenue	.17	−1.43	1.78	.79	.03	
Level of education	−1.90	−7.27	3.48	2.66	−.09	
Counseling services	−.34	−6.64	5.96	3.12	−.02	
Max facial responsiveness	3.27	−1.42	7.96	2.32	.39	
Max facial responsiveness of joy	−1.45	−3.87	.97	1.20	−.31	
Max facial responsiveness of anger	2.21	−1.58	6.00	1.88	.17	
Max facial responsiveness of surprise	1.07	−3.01	5.15	2.02	.09	
Max facial responsiveness of fear	1.33	−4.12	6.79	2.70	.10	
Max facial responsiveness of disgust	−3.26	−7.69	1.18	2.20	−.24	
Max facial responsiveness of sadness	−.16	−6.68	6.36	3.23	−.01	
Max facial responsiveness of neutral	−3.62	−8.50	1.27	2.42	−.19	
ECS total score	.21	−.16	.58	.18	.16	
DERS-F total score	.28^***^	.13	.43	.07	.55	
Step 11						.32
Constant	6.05	−3.01	15.12	4.52		
DERS-F total score	.29^***^	.18	.40	.06	.58	

*Note.* ^*^p < .05; ^**^p < .01; ^***^p < .001.

#### Chronic Stress Model Related to Dissatisfaction

In the 13th block (Table 6), the variance explained by the selected predictors was 29% [adjusted R2 = .29; F (3.52) = 8.55, *p* < .001]. The more students reported having difficulties with emotion regulation, the more they experienced chronic stress related to job and personal dissatisfaction. There was also a tendency for students showing more facial reactivity, whatever the film, to experience more chronic stress related to a lack of satisfaction (β = .21, t = 1.85, *p* = .07).

**Table 6.** Hierarchical Regression Results for Chronic Lack of Satisfaction Stress.

Variable	B	95% CI for B		SE B	β	Adjusted *R*^2^
		LL	UL			
Step 1						.21
Constant	13.38	−12.91	39.66	13.01		
Age	−.40	−1.03	.23	.31	−.21	
Gender	2.34	−5.65	10.34	3.96	.09	
Annual revenue	1.17	−.34	2.68	.75	.22	
Level of education	−3.11	−9.17	1.95	2.50	−.16	
Counseling services	2.61	−3.32	8.53	2.93	.13	
Max facial responsiveness	1.20	−3.21	5.61	2.18	.16	
Max facial responsiveness of joy	−.15	−2.42	2.13	1.12	−.03	
Max facial responsiveness of anger	.69	−2.88	4.26	1.76	.06	
Max facial responsiveness of surprise	.57	−3.26	4.40	1.90	.06	
Max facial responsiveness of fear	1.98	−3.15	7.11	2.54	.16	
Max facial responsiveness of disgust	−2.66	−6.83	1.52	2.06	−.22	
Max facial responsiveness of sadness	−.04	−6.17	6.09	3.03	−.00	
Max facial responsiveness of neutral	−3.22	−7.81	1.37	2.27	−.19	
ECS total score	.15	−.20	.50	.17	.13	
DERS-F total score	.17^*^	.03	.31	.07	.38	
Step 13						.29
Constant	8.47	−1.42	18.36	4.93		
Level of education	−3.79	−8.26	.69	2.23	−.20	
Max facial responsiveness	1.56	−.13	3.26	.84	.21	
DERS-F total score	.20^***^	.10	.31	.05	.45^***^	

*Note.* ^*^p < .05; ^**^p < .01; ^***^p < .001.

#### Model of Depressive Symptoms

In this model, 13 blocks were proposed (Table 7). The variance explained by the selected predictors was 39.4% [adjusted R2 = .39; F (3.52) = 12.92, *p* < .001]. The lower the students' emotional regulation skills (DERS-F) were, the more anger they demonstrated during angry movies, and the more likely they were to exhibit depressive symptoms. There was also a tendency for students showing less neutral facial expression when watching neutral movies to exhibit more depressive symptoms (β = −.20, t = −1.93, *p* = .06).

**Table 7.** Hierarchical Regression Results for Depressive Symptoms.

Variable	B	95% CI for B		SE B	β	Adjusted *R*^2^
		LL	UL			
Step 1						.32
Constant	1.87	−16.38	20.12	9.03		
Age	−.03	−.47	.40	.22	−.02	
Gender	.74	−4.82	6.29	2.75	.04	
Annual revenue	−.40	−1.45	.65	.52	−.10	
Level of education	−.50	−4.01	3.01	1.74	−.03	
Counseling services	1.56	−2.55	5.68	2.04	.11	
Max facial responsiveness	1.29	−1.77	4.36	1.52	.23	
Max facial responsiveness of joy	−.07	−1.65	1.51	.78	−.02	
Max facial responsiveness of anger	1.44	−1.03	3.92	1.22	.17	
Max facial responsiveness of surprise	1.43	−1.23	4.09	1.32	.18	
Max facial responsiveness of fear	−2.03	−5.60	1.53	1.76	−.22	
Max facial responsiveness of disgust	.70	−2.20	3.60	1.43	.08	
Max facial responsiveness of sadness	−.06	−4.32	4.19	2.11	−.00	
Max facial responsiveness of neutral	−2.53	−5.71	.66	1.58	−.20	
ECS total score	−.08	−.32	.16	.12	−.09	
DERS-F total score	.18^***^	.08	.28	.05	.52^***^	
Step 13						.39
Constant	−4.10	−11.18	2.97	3.53		
Max facial responsiveness of anger	2.40^*^	.60	4.19	.89	.28^*^	
Max facial responsiveness of neutral	−2.59	−5.29	.10	1.34	−.20	
DERS-F total score	.20^***^	.13	.28	.04	.61^***^	

*Note.* ^*^p < .05; ^**^p < .01; ^***^p < .001.

## Discussion

This study aimed to propose an objective measure of emotional contagion, independent from the respondent’s emotional awareness abilities, and to evaluate its association with the self-reported scale of emotional contagion, and its predictive validity. To do so, we used FACET, an automatic facial coding software using the Facial Action Coding System, to measure participants’ facial expressions as they watched movie clips eliciting specific emotional responses. As hypothesized, we found that the ECS total score was not associated with any variables related to the participants’ facial reactivity. As to our second hypothesis, facial reactivity was significantly related to personal distress, empathic concern, and depressive symptoms, even after controlling for the ECS total score, sociodemographic variables, and difficulties in emotion regulation. The ECS total score was also significantly associated with participants’ personal distress and empathic concern, but not with depressive symptoms.

### Comparison of the Study Sample with the Literature

For the ECS total score, the results of our sample appear to be slightly lower (M = 50.6) than those obtained in Doherty’s (1997) study of a sample of 226 university students (M = 54), in Wróbel and Lundqvist’s (2014) study of a sample of 633 university polish students (M = 52.3), in Rueff-Lopes and Caetano’s (2012) study sample of 1,445 Portuguese participants from the general population (M = 53.4), in Lundqvist’s (2006) study of 665 undergraduate Swedish students (M = 55.6), and in the Lo Coco et al. (2013) study of 541 young Italian adults (M = 51). This may be explained by the fact that our participants, still being students in training, have not yet had enough field experience to become aware of their own vulnerabilities as future practitioners, to know how to cope with them, and to be fully aware of their own emotions in reaction to others’, which may further highlight the limitation of self-reporting tools.

However, regarding emotional regulation (DERS-F), the results of Gratz and Roemer’s (2004) study of 357 university students documented is comparable to our study sample for both the overall score and subscales. As for empathy, our sample was compared with the study by Gilet et al. (2013) on the evaluation of the French version of the IRI among 322 French-speaking individuals (18–89 years old) in the Geneva area (personal distress M = 23.8, empathic concern M = 37.2). The mean scores of our sample appears to be comparable (respectively, M = 24.29 and M = 38.90). Finally, a slightly lower mean was noted for our study sample on the depressive symptom scale (BDI) compared to the Richards and Sanabria’s (2014) study of 254 university students (M = 11.5). Therefore, the students in our study report lower levels of distress.

### Associations between Constructs

For emotional contagion, the results of our study suggest that the two instruments, the new objective standardized measurement tool and the ECS (self-reported) do not measure the same psychosocial constructs, which means that the likelihood of showing emotional facial expressions while viewing emotionally arousing video stimuli is not associated with vulnerability to emotional contagion, as measured by the ECS using participants’ self-reported measures. This result is like that of studies on the perception and objective measurement of stress. The meta-analysis of Campbell and Ehlert (2012), for example, concludes that only about a quarter of the research analyzed has significant associations between objective responses and participants’ self-reported measures. The weakness of the relationship between objective responses and self-reported measures can be attributed to many factors. For example, some self-reported scales have low construct validity, which may result in a discrepancy between subjects’ subjective and objective responses. In addition, the individual characteristics of the participants, such as their personality, emotional regulation ability, and level of social desirability can lead to discrepancies between their subjective and objective responses for the same constructs (Campbell & Ehlert, 2012). In our study, discrepancies may also be attributed to the effect of the context. For the self-reported responses, students may have reported their vulnerability to emotional contagion by mentally putting themselves in a helping situation, whereas our objective task measures their vulnerability through a playful task, that is, viewing movie clips.

It should be noted that our participants were still university students, so they may not have had enough experience to be aware and accurately assess their vulnerability to the emotions of others. In the field of helping relationships, the distinction between self and others is a well-known training challenge (Wispe, 1986), and it is highly influenced by people’s experience and perception of interoceptive changes (Tajadura-Jimenez & Tsakiris, 2014; Tsakiris et al., 2011). Therefore, if the variability in interoceptive accuracy, and thus emotional awareness, had been higher in our sample, the objective tool would have been a more accurate measure than the self-report tool.

The ECS had a good internal consistency in our sample (α = .80). As already observed in other studies, it was significantly higher for women than for men (Rueff-Lopes & Caetano, 2012; Wróbel & Lundqvist, 2014). It was also significantly associated with the participants’ levels of empathic concern, and personal distress (r_119_ = .37 and r_119_ = .31, respectively), as already noticed in Doherty’s original paper. However, the ECS total score was not related neither to chronic stress, nor to depressive symptoms in our study.

Our proposed measures of facial responsiveness also correlated significantly with personal distress and empathic concern. The more participants displayed neutral facial expression during neutral films, the more they reported experiencing personal distress. Neutral video clips were ranked 4th and 16th (last) among video stimuli and are relatively briefer than the others. Neutral facial responsiveness in this context may be interpreted as the use of suppressive emotional expression as a strategy of emotional regulation since participants did not know the emotional nature of each video clip, and they may expect emotional content. Association between the use of suppressive expression and adverse social outcomes has already been reported (Srivastava et al., 2009). Another hypothesis may be that neutral expression could also have some residues of past emotions (Albohn & Adams, 2020) not detected by the algorithms.

As to empathic concern, displaying more facial expression throughout the presentation of the emotional video clips was related to less empathic concern. This result may be surprising since the self-reported vulnerability to emotional contagion was positively related to empathic concern (and personal distress). In a study with 281 Salvation Army officers using the Interpersonal Reactivity Index to assess empathy and the Maslach Burnout Inventory for occupational mental health, Gross (1994) found that personal distress and empathic concern were, respectively, negatively and positively associated with a sense of efficacy. They also found that personal distress was a significant predictor of emotional exhaustion when empathic concern appeared to be a significant protector against depersonalization. This is consistent with other findings showing that empathic concern is often related to social and healthcare professionals’ satisfaction and mental health (Lamothe et al., 2014; Paro et al., 2014; Shanafelt et al., 2005), while personal distress is associated with burnout and compassion fatigue (Thomas, 2013). Considering that empathic concern may be seen as a protective factor in healthcare providers’ mental health, it is not surprising that being more emotionally reactive (thus more facially responsive) to the videos eliciting emotions was related to less empathic concern even when controlling for the self-reported vulnerability to emotional contagion. This result further suggests that an objective measure better accounts for the adverse effects of unconscious emotional contagion in our participants. More specifically, participants may be aware of their vulnerability to others' emotions, and thus may use this awareness to increase empathic concern, while experiencing the adverse effects of nonconscious vulnerability to others' emotions.

Moreover, it is not surprising to find that only facial reactivity remains positively correlated with participants’ depressive symptoms. Indeed, when viewing movie clips of anger, the more a participant exhibited a spike of anger in facial reactivity and the less they exhibited neutral facial expressions when watching neutral movie clips, the higher they scored on the depressive symptoms scale. This suggests that in our study sample, being reactive to the emotions of others, specifically to anger, and having difficulties coming back to a neutral state (which translates in difficulties regulating one’s emotions), would be associated with a greater risk of exhibiting depressive symptoms. Interestingly, such association has already been reported between a self-reported measure of revised 5-item ECS and the level of depression in 751 social workers (mean age 44 y.). The strength of the association found in this study is close to the one we found (r_751_ = .33) with our objective measure of emotional contagion (Siebert et al., 2007). It is possible that over the years, as social workers gain more emotional awareness, they will be better at assessing emotional contagion than our students, who are still in training and who have limited field experience. Those results are also in line with the Joiner and Katz (1999) meta-analysis showing that depressive symptoms are contagious. Furthermore, anger and mood disorders are known to be related (Cassiello-Robbins & Barlow, 2016), and one can easily imagine that social workers are in contact with people living lots of negative emotions including irritation and anger, and that social workers could be easily contaminated by such emotions, explaining why people highly vulnerable to anger could be more at risk of depressive symptoms. Not to mention, that Petitta et al. (2017) have shown that emotional contagion of anger is related to doctors’ risk of emotional exhaustion.

The difficulties in the emotion regulation scale contributed to the dependent outcomes in our study, as found in previous studies: personal distress (Grynberg & Lopez-Perez, 2018), chronic stress (Katz et al., 2018) and depression (Thomas, 2013; Welkie et al., 2021). On the other hand, empathic concern was not found to be related to difficulties in emotional regulation. This shows once again the particularity of this construct and the benefits for the well-being of workers in helping professions. Finally, the high associations between emotional regulation scores (DERS-F) and those of the depressive symptoms (BDI) and stress-dependent variables (TICS) highlight the importance of emotional regulation and its central role in mental health risks for our sample of social work students. This is hardly surprising. Because emotional regulation is a key component of psychological health (Aldao et al., 2010), it has been argued that high levels of depressive symptoms and job stress can result in a lack of effective emotional regulation strategies (Gross & Munoz, 1995; Troy et al., 2010).

No association was found between our measures of emotional contagion and the dimensions of chronic stress reported by our participants. This result is surprising since previous studies have found that stress is highly contagious (Buchanan et al., 2012; Engert et al., 2014). Also, one could expect a significant association between a measure of vulnerability to emotional contagion and chronic stress considering the high levels of stress students are usually experiencing (Linden & Stuart, 2020). Nevertheless, we noted that students tended to show more facial reactivity, whatever the movie clips, and to experience more chronic stress related to a lack of satisfaction, but the non-significance of this association may result from a lack of statistical power. Only emotional regulation difficulties would predict 32% of the variance in high demand chronic stress. The link between emotional regulation difficulties and stress is not surprising. Several studies have identified the use of effective regulation techniques as being associated with lower levels of stress and higher levels of well-being (Buruck et al., 2016; Katana et al., 2019).

Since this is a cross-sectional correlational research design, results about depressive symptoms could be interpreted differently: depression leading to less facial expressivity. In this case, our result goes against the emotional context insensitivity hypothesis, which suggests that depressed people show little emotional response in both positive and negative contexts (Rottenberg et al., 2005). These individuals’ depressed state would then influence the way they react to environmental changes around them and make them inhibit themselves. It should be noted, however, that the severity and chronicity of the depressive state are two factors that can influence a person’s emotional reactivity (Allen et al., 1999). In other words, the emotional response of inhibition would be more present in individuals with more severe and chronic symptoms of depression compared to a population demonstrating few or no symptoms, as is the case for our study sample. Moreover, according to the BDI clinical thresholds Beck et al. (1988), 16 students in our sample exceed the minimal threshold for depressive symptoms, and of these, seven exceed the moderate depression threshold.

Although our objective measures of emotional contagion seem to predict the risk of developing depressive symptoms among this study’s participants, results should be interpreted with caution. The study was carried out using a cross-sectional design, which does not determine the causal links or mechanisms explaining the results obtained. And since the data were only collected at one point in time, they only reflect the characteristics of the sample at a specific point in time. In addition, having a small sample size inevitably has an impact on the statistical power of the study. With a small sample size, it is difficult to generalize the results obtained from a specific population. Finally, the variability in the duration of stimuli presented may have introduced certain bias. However, results were still significant with facial reactivity to anger-eliciting movie-clips, which are not the longest stimuli presented. Therefore, replicating this research, with a longitudinal design and a larger number of participants, would allow for a better understanding of the observed effects.

## Conclusion

Our study suggests that the two measures of emotional contagion – objective and self-reported – are not related. This result, and more precisely the association found with empathic concern and depressive symptoms, suggest that our objective measure can capture the automatic and unconscious processes of emotional contagion which, if not regulated, can result in severe consequences. Considering that our results also point toward an effect of unconscious emotions, one potential way to address this issue for social workers students would be to offer interventions promoting emotional awareness (Lane & Smith, 2021; Yogeswaran & El Morr, 2021), but also awareness towards others’ emotions such as programs dedicated to increase interpersonal accuracy (Schlegel et al., 2017).

## Supplemental Material

Supplemental Material - Facial Reactivity to Emotional Stimuli is Related to Empathic Concern, Empathic Distress, and Depressive Symptoms in Social Work Students Supplemental Material for Facial Reactivity to Emotional Stimuli is Related to Empathic Concern, Empathic Distress, and Depressive Symptoms in Social Work Students by Pierrich Plusquellec, Kaylee Smart, Vincent Denault in Psychological Reports
