# Supplementary material for: Facial Reactivity to Emotional Stimuli is Related to Empathic Concern, Empathic Distress, and Depressive Symptoms in Social Work Students
Source: Psychol Rep. 2023 Jul 3;128(4):2304–35. doi: 10.1177/00332941231181027 (PMC12149470; doi:10.1177/00332941231181027)
Supplement: Supplemental Material - Facial Reactivity to Emotional Stimuli is Related to Empathic Concern, Empathic Distress, and Depressive Symptoms in Social Work Students [file sj-pdf-1-prx-10.1177_00332941231181027.pdf]

Supplementary materials

Table S1
Film clips used to create the objective emotional contagion tool

Assignment order	Movie title	Duration (seconds)	Elicited emotion
1.	*Benny & Joone*	121	Joy
2.	*City of Angels*	255	Sadness
3.	*The Shining*	255	Fear
4.	*Blue 3*	25	Neutral
5.	*Child's Play – Chucky*	65	Surprise
6.	*Saving Private Ryan*	322	Disgust
7.	*Les Trois Frères*	129	Joy
8.	*Schindler's List 2*	115	Anger
9.	*Trainspotting 3*	62	Surprise
10.	*Le Dîner de Cons*	100	Joy
11.	*Schindler's List*	78	Disgust
12.	*Les Visiteurs*	129	Joy
13.	*Dangerous Minds*	128	Sadness
14.	*Scream 1*	393	Fear
15.	*Sleepers*	140	Anger
16.	*Blue 1*	16	Neutral

The following link provides access to the film clips used to create the emotional contagion objective measurement task:
https://sites.uclouvain.be/ipsp/FilmStim/film.htm
